# Celebrating student engagement in an undergraduate histology course: A showcase review

**DOI:** 10.1002/ase.70011

**Published:** 2025-02-24

**Authors:** Kayla Vieno‐Corbett, Andrew M. Deweyert

**Affiliations:** ^1^ Department of Anatomy and Cell Biology, Schulich School of Medicine & Dentistry Western University London Ontario Canada

**Keywords:** educational innovation, histology, review session, STEAM, student engagement, undergraduate education

## Abstract

Drawing is a teaching tool that provides numerous benefits to student learning, including enhanced knowledge retention, improved observation skills, and increased engagement with course content. However, these exercises also place high cognitive demands on students and require a considerable time commitment. To acknowledge and celebrate the effort students invest in their drawings; while also giving these illustrations curricular significance, a gallery walk can be an effective teaching strategy. During a gallery walk, students move around a learning space to view, analyze, and discuss work displayed on the walls. This article describes an adaptation of a gallery walk, named a ‘showcase review session’, which was implemented in an undergraduate histology course. This optional session highlighted highly accurate assignment drawings while offering a content review before the final examination. The course instructor created review questions associated with the student drawings, which were projected onto screens around the room. Students could visit the session at any time, with the questions cycling continuously, and the course instructor and teaching assistants circulated to answer questions. Students responded positively to the session, noting that it helped them prepare for their upcoming practical laboratory examination and that showcasing student work added value to the course. Showcase review sessions like the one described can be applied across disciplines, giving students the opportunity to learn from their peers' work while effectively reviewing course content in an engaging and interactive environment.

## INTRODUCTION

Histology is a complex subject that requires students to identify and interpret intricate cellular and tissue structures, often involving a vast amount of material. This complexity can overwhelm students, leading to challenges in knowledge retention and application.^1^ Additionally, students may perceive histology as less interesting compared to more clinically oriented subjects, resulting in reduced motivation and engagement.^1^ Creating labeled drawings of observations made through a light microscope is a traditional teaching tool used in histology courses that can be an effective strategy for addressing these challenges.^1^, ^2^ This approach has been shown to enhance long‐term knowledge retention, foster engagement by encouraging higher‐order thinking, and even improve performance on practical assessments.^3^, ^4^, ^5^ However, despite these benefits, students may be reluctant to fully embrace drawing exercises due to the associated cognitive demand and time commitment.^6^

A gallery walk is an active learning strategy that can be used to celebrate the effort students invest in their histological drawings or other coursework. In a gallery walk, students rotate through stations featuring materials, questions, or prompts, often working collaboratively in groups.^7^, ^8^, ^9^ While not always the case, some gallery walks are designed to display student work at the stations.^10^, ^11^, ^12^ This strategy has been shown to enhance engagement and foster higher‐order thinking by encouraging students to synthesize and apply content learned during lessons.^10^, ^13^, ^14^ Studies collecting student perceptions of gallery walks report that they appreciated these sessions, finding them valuable to their overall learning, promoting collaboration, and encouraging active participation.^10^, ^15^, ^16^ Furthermore, two studies found that students who participated in gallery walk sessions achieved higher test scores compared to those taught using traditional didactic lectures.^14^, ^17^ Gallery walks offer an interactive approach to sharing students' work, providing an opportunity to recognize the significant cognitive effort students invest in their coursework while fostering an engaging learning environment.

Although gallery walks are described in the literature across various post‐secondary disciplines, their application in the anatomical sciences appears to be limited. However, a gallery walk could be particularly suitable for a histology course as it fosters active engagement, collaboration, and discussion around complex content, such as accurate interpretation of microscopic images. By incorporating visual elements like drawings and illustrations, this strategy aligns with the STEAM (Science, Technology, Engineering, Arts, Mathematics) teaching approach, which emphasizes integrating the arts into traditional STEM (Science, Technology, Engineering, Mathematics) fields.^18^, ^19^ This approach combines STEM's analytical focus with the creativity and design principles of the arts, encouraging students to think deeply and critically about scientific concepts. Incorporating artistic elements into histology education is further supported by dual‐coding theory, which explains that individuals can encode both verbal and visual mental models of learned content, as well as connections between them.^20^, ^21^ Consequently, information is more effectively retrieved when represented both verbally and visually, such as through drawings.^20^, ^21^ Gallery walks can address specific challenges in histology education, such as improving the retention of visually dense information and bridging the gap between theoretical knowledge and practical microscope usage.

Although gallery walks are not a prominent topic in current anatomical sciences literature, other active learning strategies have been widely supported in histology courses. These strategies include incorporating active learning PowerPoint slides into traditional lectures,^22^ team‐based active learning laboratory exercises,^23^, ^24^ case‐based seminars,^25^ interactive computer‐based laboratory manuals,^26^ and gamification tools.^27^, ^28^ All of these strategies share a common goal of transitioning away from passive lecture environments to more learner‐centered experiences, thereby enhancing learning efficacy. Evidence consistently shows that active learning strategies yield positive outcomes in histology education.^22^, ^23^, ^24^, ^25^, ^26^, ^27^, ^28^ Additionally, the gallery walk has been successfully implemented in other post‐secondary STEM disciplines, including medicine, nursing, and undergraduate science.^8^, ^13^, ^14^ STEM disciplines often require mastery of foundational concepts before progressing to advanced topics. Gallery walks offer an opportunity for students to synthesize these concepts while developing higher‐order thinking skills, such as analysis, evaluation, and synthesis, which are important in STEM education.^8^

An adaptation of a gallery walk was implemented in a third‐year undergraduate histology course to showcase students' assignment drawings and provide an optional review session. An overview of this optional showcase review session is presented below, preceded by a description of the course in which it was offered and the assignments from which it was developed.

## DESCRIPTION

### Overview of course

The showcase review session comprised part of a third‐year mammalian histology course, predominantly for students enrolled in the Bachelor of Medical Sciences degree program. A blended instructional model offered both in‐person and online lectures and laboratories, allowing students to choose the combination that aligned with their schedules and learning preferences. The 2023–2024 offering of the course had 299 students enrolled.

The mammalian histology course offered a detailed study of the cellular and microscopic structure of the various tissues and organ systems of the body, with emphasis on humans and other mammals used in medical research. Systems were examined, stressing the relations of structure to function. The course objectives include:

- Describe how tissues and organs are structured to perform various functions.
- Identify normal tissues and organs at the microscopic level.
- Integrate knowledge gained from this course with your other life science courses.

### Overview of laboratory assignments

The assignments were designed and implemented to maintain student engagement and facilitate progress through the course material. The widely recognized concept that “assessment drives learning” informed the decision to include these assignments, ensuring students stay on track.^29^ Histology is a cognitively dense subject, and the curriculum is scaffolded to begin with cellular structures, progress to tissues, and conclude with organs and organ systems. As such, these assignments assist with mastery of lower‐order foundational material, which is essential for comprehension of advanced content as the course progresses.

**FIGURE 1:**
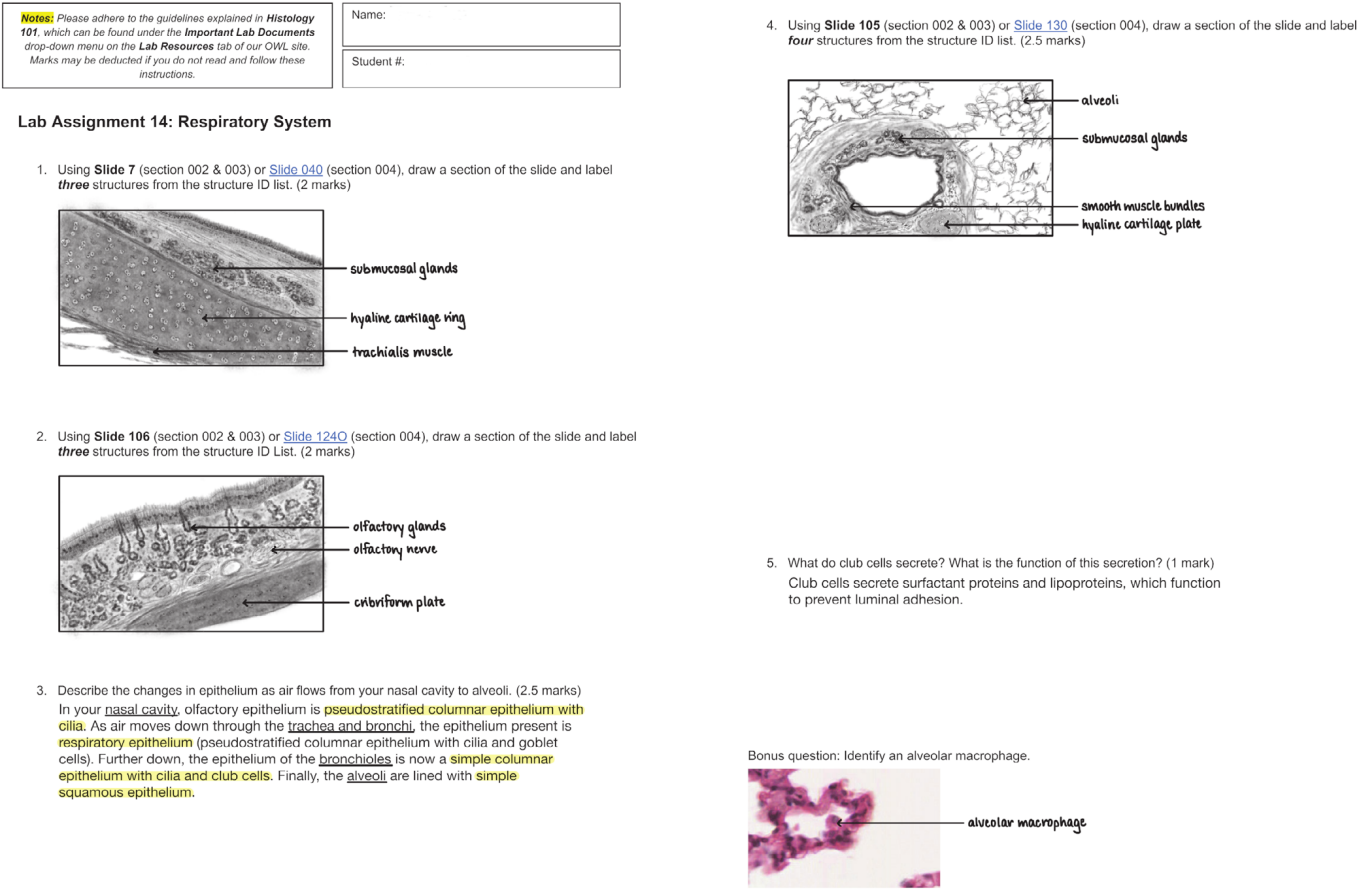
Sample of a completed laboratory assignment from an undergraduate mammalian histology course. This assignment was completed by Kaite Winsor, a student in the 2023–2024 offering of the course.

Each laboratory assignment was composed of five questions plus an additional bonus question. The question formats included drawing and short answer. Drawing questions asked students to view a specific slide using the physical or virtual microscope and draw and label a section of the tissue or organ they were observing. Students could complete their drawings on paper or using digital software, with illustrations in color or black and white. Figure 1 illustrates an example of a completed laboratory assignment.

### Overview of optional showcase review session

**FIGURE 2:**
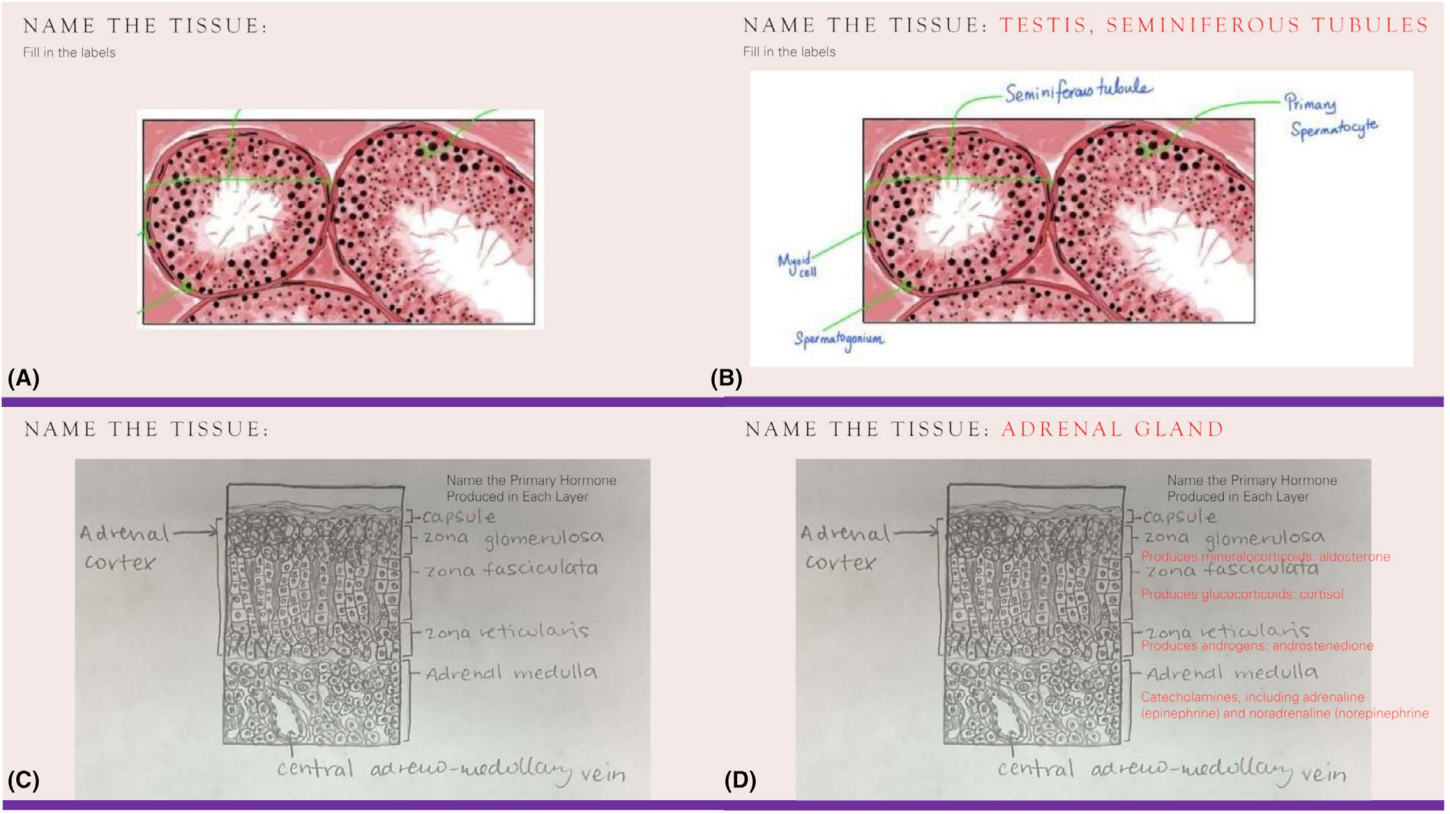
Sample questions from an optional showcase review session. (A) A review question showcasing a student illustration created using digital drawing software. This drawing was completed by Leena Mohamed Faleel, a student in the 2023–2024 offering of the undergraduate mammalian histology course. (B) The answer slide for the review question illustrated in (A). (C) A review question showcasing a student illustration created using pencil and paper. This drawing was completed by Isabel Lewin, a student in the 2023–2024 offering of the undergraduate mammalian histology course. (D) The answer slide for the review question illustrated in (B).

The teaching team discovered that many student drawings created for assignments were at the level of medical illustrations, which inspired the creation of the optional showcase review session to celebrate students' work. Throughout the course, as assignments were graded, teaching assistants collated student drawings that accurately illustrated the key features of the various cells, tissues, and organs. The course instructor used these drawings to create the optional showcase review session. To do this, the instructor generated a PowerPoint presentation that anonymously displayed students' drawings along with associated practice questions representative of the final course examination. In total, 19 review questions were created. Question formats included labeling and short answer, with a focus on structure identification and connection to theoretical knowledge such as tissue functions or physiological processes. Figure 2 illustrates several slides from the showcase review session PowerPoint presentation.

The showcase review session was aligned with the course's learning objectives, which include identifying tissues and organs microscopically and understanding their structure–function relationships. It is important to emphasize that the selection of drawings for the showcase review session was not based on artistic skill. From the start of the semester, students were informed that the primary focus of their drawings should be on accurately representing the key features of the cells, tissues, or organs they observed under the microscope. For example, if students were asked to draw a gastric gland in the stomach, it was expected that the identifying features of each cell type would be accurately depicted. Parietal cells should be large and round; chief cells should be granular; and mucous cells should have an uncolored cytoplasm. Focusing on key histological features during the drawing process fosters critical thinking and analytical skills essential for assessments like quizzes and bell‐ringer exams.^30^, ^31^

Student drawings included both color and black‐and‐white examples in the review session. While color can aid in distinguishing histological components, black‐and‐white images challenge students to rely on shape and structure, fostering a deeper understanding of tissue organization. This approach is particularly valuable for accommodating students with color vision deficiencies, as visual impairments like these present an ongoing accessibility challenge in histology education.^32^, ^33^, ^34^ The course also emphasizes integrating histological knowledge with content from other life science courses. The collaborative nature of the review session supported this objective, encouraging peer learning and exposing students to diverse perspectives and educational backgrounds.^23^ Review questions focusing on physiological processes or functions further facilitated the integration of histological interpretation with broader life science concepts.

This session functioned as a formative assessment that prepared students for their practical laboratory summative assessment. The showcase review session took place one week before the course's final cumulative examination, anticipating that this timing would boost students' motivation to attend. This optional learning opportunity consisted of two drop‐in sessions, each lasting two hours. The teaching team projected the PowerPoint presentation onto multiple screens around the laboratory space, running it on a continuous cycle of one minute per question, with the answers appearing afterward. During this time, students could work through the showcase at their own pace, independently or in groups, and the course instructor and several teaching assistants were available to answer questions.

## DISCUSSION

A novel aspect of the showcase review session was the integration of student‐generated content, repurposing assignment submissions to serve an additional role within the curriculum. Although the class was informed in advance that anonymous student drawings might be used—with an opt‐out option via email—they did not know whether their own illustrations would be selected for display. Seeing one's work included in the session could foster a sense of connection to the material, increasing emotional engagement with the content.^35^ Using student‐generated content also provided active learning benefits. Incorporating drawings introduced multiple representations, transitioning the session away from traditional histological diagrams and micrographs to a more learner‐centered modality. Research indicates that providing multiple means of representation in anatomical sciences reduces cognitive load and enhances learning environments, addressing the common challenge of knowledge retention in histology.^1^, ^36^ Further, deciphering peer‐generated work in a collaborative environment challenges students to think critically and engage in peer instruction, a strategy shown to increase student engagement and improve accuracy in subsequent related work.^37^, ^38^ Given the challenges of engagement and knowledge retention in histology education, this approach encourages students to refine their understanding by articulating and defending their reasoning, thereby fostering deeper involvement with, and mastery of, course concepts. Implementing student‐generated content into review sessions can effectively enhance student engagement, active learning, and collaborative peer instruction.

Approximately one‐third of the students enrolled in the course participated in the optional showcase review session (~100 students). Quality improvement feedback for this session was collected through an anonymous survey at the end of the academic year (*n* = 16). The overall experience with the showcase review session was rated as excellent or good by 83.3% of attendees. Students highlighted several positive aspects of the session, noting that the session was well‐organized and effectively implemented, the questions were representative of the exam, and the opportunity to ask the teaching assistants and professor questions in real time was beneficial. Students offered constructive suggestions for improvement, including adding more questions for additional practice, ensuring question clarity, and incorporating a station attended by a TA or professor for reviewing specific questions. These suggestions are practical and can be integrated into future iterations of the showcase review session to enhance its effectiveness. Overall, students responded positively to this pilot session, identifying several benefits to their learning and engagement.

The success of the novel showcase review session demonstrates the potential of innovative pedagogical applications in histology education, particularly those integrating multimodal learning strategies. Many students in the course were high achievers aspiring to professional degrees and frequently requested additional review and support materials. The showcase review session not only celebrated students' work but also demonstrated how their assignment drawings could serve as effective study tools, enhancing their understanding and retention of histological concepts. In addition to addressing challenges in histology education, such as knowledge retention and content complexity,^2^ the showcase review session provided opportunities for peer interaction and collaboration, fostering a sense of community and social engagement.^39^ However, due to logistical constraints, gallery walks would be better suited as a complementary strategy rather than a replacement for lecture‐based teaching. Sessions like the showcase review described here exemplify how active learning approaches can effectively supplement traditional methods to enhance student learning and engagement.

As this showcase review session served as a pilot, a few challenges were encountered. Although the course employed a blended instructional model with both in‐person and online lectures and laboratories, only an in‐person format was used for the showcase review session. This format provided remote students with a valuable opportunity for in‐person interaction with their peers and the teaching team. The sessions took place during the typical laboratory times for in‐person sections, which could have posed challenges for online students who chose the virtual format due to potential scheduling conflicts. However, the teaching team deemed it infeasible to transition the session to an online format in its inaugural year, given its purpose and organization, and considering that it was not the only review session offered in the course. An additional challenge in creating the showcase review session was the workload placed on the instructor. While students produced the drawings, the teaching team still needed to choose those for inclusion in the session, and the instructor had to develop the questions. Since the session's goal was to highlight student work, the PowerPoint presentation will require annual updates for each new cohort. To ease the instructor's workload, teaching assistants selected accurate drawings while grading assignments throughout the year. This approach allowed the creation of the showcase review session to be an ongoing process rather than a single, substantial task at the end of the course. Furthermore, now that the inaugural session has been developed, the same questions can be reused each year, with only the drawings updated to reflect the current cohort's work. Despite these challenges, the teaching team believed that the benefits of the session and student buy‐in outweighed the additional work.

The authors acknowledge both short‐ and long‐term directions for the future development of the showcase review session. In the short term, the teaching team aims to transition the session to an online format to enhance accessibility for all learners. Based on student feedback, the session will also be expanded to include additional questions, with a focus on ensuring the clarity and alignment of the questions with course content. In the in‐person format, a dedicated station will be added where students can review specific questions with a teaching assistant, eliminating the need to wait for the question to reappear in the next cycle. In the long term, the authors plan to continue evaluating the impacts of the showcase review session, making revisions and updates as necessary. While this session was initially implemented in an undergraduate histology course, the authors believe this teaching method could be successfully adapted by instructors across other STEM disciplines that emphasize illustration or involve frequent assignments.

The teaching team developed the optional showcase review session as a gallery walk adaptation to celebrate the effort students invested in their assignment drawings throughout the course, while also providing a content review before the final examination. The session was well received by students, who provided positive feedback on its effectiveness in aiding their preparation for the final examination. This approach allowed students to learn from their peers' work while creating an engaging and interactive environment for effective content review. Implementation of showcase review sessions in histology education and other STEM disciplines can promote engagement, peer‐to‐peer learning, and critical thinking.

## AUTHOR CONTRIBUTIONS

**Kayla Vieno‐Corbett:** Conceptualization; writing – original draft; writing – review and editing. **Andrew M. Deweyert:** Conceptualization; writing – review and editing; supervision.

## CONFLICT OF INTEREST STATEMENT

The authors have no conflicts of interest to disclose.

## ETHICS STATEMENT

Ethics approval was not required for this article as it did not involve direct data collection or interaction with human participants. See https://ethics.gc.ca/eng/tcps2‐eptc2_2018_chapter2‐chapitre2.html.
